# High-dose atorvastatin therapy progressively decreases skeletal muscle mitochondrial respiratory capacity in humans

**DOI:** 10.1172/jci.insight.174125

**Published:** 2024-02-22

**Authors:** Terence E. Ryan, Maria J. Torres, Chien-Te Lin, Angela H. Clark, Patricia M. Brophy, Cheryl A. Smith, Cody D. Smith, E. Matthew Morris, John P. Thyfault, P. Darrell Neufer

**Affiliations:** 1 East Carolina Diabetes and Obesity Institute and; 2 Department of Physiology, Brody School of Medicine Greenville, North Carolina, USA.; 3 Department of Kinesiology, East Carolina University, Greenville, North Carolina, USA.; 4 Cell Biology and Physiology and; 5 Kansas University Diabetes Institute and Department of Internal Medicine, Division of Endocrinology, University of Kansas Medical Center, Kansas City, Kansas, USA.; 6 Department of Biochemistry and Molecular Biology, Brody School of Medicine, Greenville, North Carolina, USA.

**Keywords:** Muscle biology, Bioenergetics, Mitochondria, Skeletal muscle

## Abstract

**BACKGROUND:**

While the benefits of statin therapy on atherosclerotic cardiovascular disease are clear, patients often experience mild to moderate skeletal myopathic symptoms, the mechanism for which is unknown. This study investigated the potential effect of high-dose atorvastatin therapy on skeletal muscle mitochondrial function and whole-body aerobic capacity in humans.

**METHODS:**

Eight overweight (BMI, 31.9 ± 2.0) but otherwise healthy sedentary adults (4 females, 4 males) were studied before (day 0) and 14, 28, and 56 days after initiating atorvastatin (80 mg/d) therapy.

**RESULTS:**

Maximal ADP-stimulated respiration, measured in permeabilized fiber bundles from muscle biopsies taken at each time point, declined gradually over the course of atorvastatin treatment, resulting in > 30% loss of skeletal muscle mitochondrial oxidative phosphorylation capacity by day 56. Indices of in vivo muscle oxidative capacity (via near-infrared spectroscopy) decreased by 23% to 45%. In whole muscle homogenates from day 0 biopsies, atorvastatin inhibited complex III activity at midmicromolar concentrations, whereas complex IV activity was inhibited at low nanomolar concentrations.

**CONCLUSION:**

These findings demonstrate that high-dose atorvastatin treatment elicits a striking progressive decline in skeletal muscle mitochondrial respiratory capacity, highlighting the need for longer-term dose-response studies in different patient populations to thoroughly define the effect of statin therapy on skeletal muscle health.

**FUNDING:**

NIH R01 AR071263.

## Abstract 2

<p>Eight weeks of high dose atorvastatin treatment decreased skeletal muscle mitochondrial functional capacity in overweight but otherwise healthy humans.</p>

## Graphical abstract

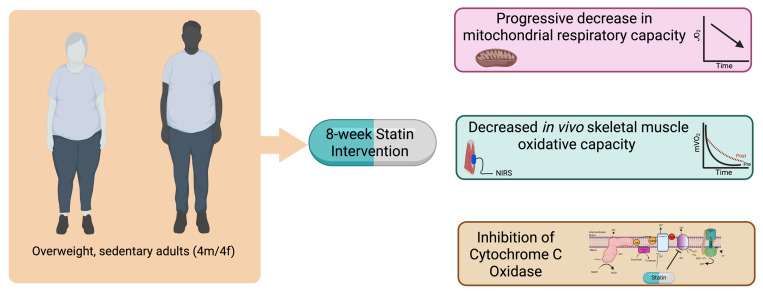

## Introduction

Statins are highly effective at lowering circulating low-density lipoprotein (LDL) cholesterol and promoting both primary and secondary prevention of atherosclerotic cardiovascular disease (ASCVD) (1–3). While statin therapy is generally well tolerated and considered safe (1), randomized control trials and large cohort studies, together with patient registries, report that up to 30% of patients experience mild to moderate muscle weakness, pain, and/or cramps (4–11), often leading to nonadherence and discontinuation of statin use, thus adversely affecting ASCVD outcomes (12, 13).

The potential mechanism underlying the pathophysiology of statin intolerance remains unclear. While rare, the most severe form of statin intolerance is characterized by serum creatine kinase increasing to more than 10 times the upper normal limit due to release from damaged muscle (i.e., rhabdomyolysis; 2–3 in 100,000 per year), jeopardizing renal function within days of initiating statin therapy (14). The response typically occurs in patients taking other high-risk medications and/or with other comorbidities (15, 16), implying a preexisting susceptibility to collapse of muscle cell function. By contrast, the myopathic symptoms characteristic of the more common milder form of statin intolerance occur with little to no increase in creatine kinase (17). The risk of developing myopathic symptoms increases with age as well as dose and duration of statin therapy (10, 18, 19), suggesting a progressive deterioration in one or more aspects of muscle function. Moreover, several studies have provided evidence that, when statins are combined with regular exercise, the prevalence and severity of adverse muscle reactions increase (20–25) and the cardiovascular benefits associated with exercise training may be attenuated (26–28).

Mounting evidence has emerged that mitochondrial function may be a primary target of statins. In vitro exposure of isolated muscle mitochondria, permeabilized myofibers, and/or cultured myocytes to statins has been shown to dose-dependently inhibit the enzymatic activity of respiratory complexes and overall mitochondrial respiratory capacity, and it has been shown to disrupt calcium homeostasis (29–34). The effect of in vivo exposure to statins on muscle respiratory function in humans, however, is less clear. In a study of patients with hypercholesterolemia treated with simvastatin (10–40 mg/d) for at least 12 months (average 5 years), maximal ex vivo respiratory capacity measured in permeabilized muscle fiber bundles was significantly lower compared with fibers from well-matched controls (35). However, in a subsequent well-controlled study of healthy patients treated with statins (simvastatin 80 mg/d or pravastatin 40 mg/d) for 2 weeks, only a trend for a decrease in muscle mitochondrial respiratory function was detected (36). Together, these 2 studies suggest the effect of statins may be relatively slow and progressive. To test this possibility, we examined the effect of high-dose (80 mg/d) atorvastatin therapy over 8 weeks on skeletal muscle mitochondrial function and whole-body aerobic capacity in overweight but otherwise healthy humans. The findings revealed a striking progressive decrease in mitochondrial respiratory capacity in skeletal muscle.

## Results

### Short-term high-dose statin therapy reduces muscle respiratory capacity and insulin sensitivity.

To directly measure the potential effect of high-dose statin therapy on whole body and skeletal muscle aerobic function, 8 overweight but otherwise healthy sedentary adults (4 male and 4 female) were studied before and 14, 28, and 56 days after initiating atorvastatin (80 mg/d) therapy. Participant physical and clinical characteristics are presented in Table 1. As expected, statin therapy significantly reduced circulating LDL (47%) and total (32%) cholesterol. Creatine kinase, a marker of muscle damage, tended (*P* = 0.072) to increase after 56 days of atorvastatin treatment to 149.5 ± 90.0 U/L but remained well below the upper normal limit (336 U/L) in all patients. Total bilirubin and alkaline phosphatase, 2 markers of liver damage, increased significantly from baseline (Table 1) but also remained well below their respective upper normal limits (1.2 mg/mL and 147 U/L). None of the patients reported myalgia or any other muscle-related symptoms throughout the study.

**Figure 1:**
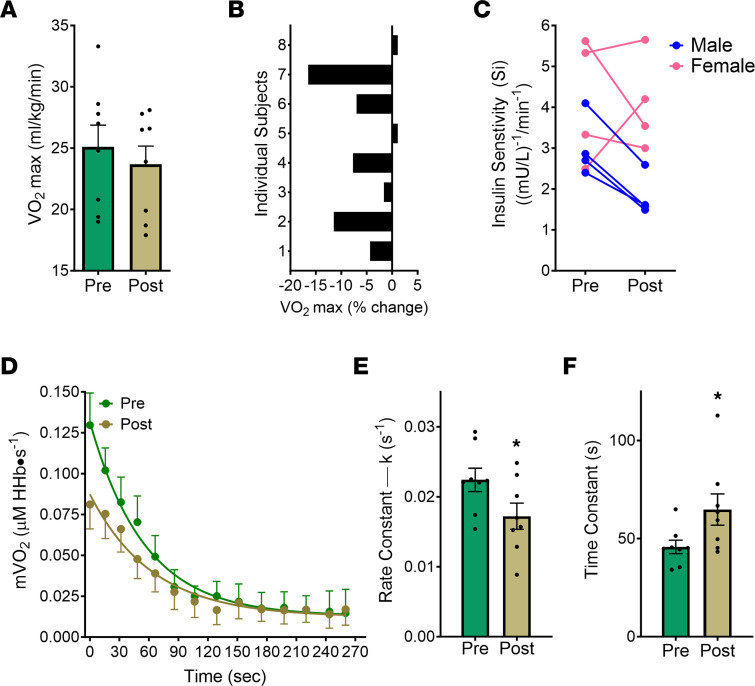
Short-term statin therapy decreases skeletal muscle mitochondrial function in vivo and aerobic capacity. (**A**) Whole-body aerobic capacity (VO_2_ max) before and after 56 days of statin therapy. (**B**) Relative individual changes (%) in VO_2_ max after statin therapy. (**C**) Insulin sensitivity measured by IVGTT. (**D**) Postexercise recovery kinetics of muscle oxygen consumption measured by near infrared spectroscopy (mVO_2_) before (pre) and after (post) 56 days of statin therapy. (**E** and **F**) Following a single exponential fitting, the calculated rate constants (directly related to mitochondrial capacity) (**E**) and time constants (inversely related to mitochondrial capacity) (**F**) are shown. Data are mean ± SEM (*n* = 8 for all panels). Data analyzed by paired *t* tests. *P < 0.05 was considered significant.

Whole-body maximal aerobic capacity (VO_2_ max), as assessed by indirect calorimetry during a graded treadmill test, was not significantly affected by 8 weeks of atorvastatin therapy (Figure 1A). Individual responses to statin therapy varied, however, with 3 of 8 patients showing no change or a slight increase (+1.7 mL/kg/min [+1.1%]) and 5 of 8 showing a decline in VO_2_ max ranging from –0.9 to –5.5 mL/kg/min (Supplemental Figure 1; supplemental material available online with this article; https://doi.org/10.1172/jci.insight.174125DS1) or –4.3 to –16.5% (Figure 1B).

Given that previous large cohort studies (37) and metaanalyses (38–40) have provided evidence that statin therapy may increase the risk of developing insulin resistance/diabetes, i.v. glucose tolerance tests (IVGTT) were performed at baseline and at the end of the statin therapy. Six of the 8 participants displayed a reduction in insulin sensitivity after 56 days of atorvastatin therapy, while 2 participants (both female) unintentionally lost weight (3–5 kg) and exhibited a slight to modest increase in insulin sensitivity (Figure 1C).

To measure aerobic capacity specifically in skeletal muscle in vivo, noninvasive near-infrared spectroscopy (NIRS) was employed (41–44). Briefly, following a short period of contraction of the vastus lateralis muscle sufficient to increase muscle oxygen consumption (mVO_2_) by approximately 7- to 10-fold, the recovery kinetics of mVO_2_, which is directly related to the overall aerobic capacity of the tissue, was measured during a series of arterial/venous occlusions (Figure 1D). mVO_2_ kinetics during recovery decreased after 56 days of atorvastatin treatment, as evidenced by an approximately 23% decrease in the rate constant (*k*) and an approximately 45% increase in the time constant (τ = 1/*k*) for ΔmVO_2_ (Figure 1, E and F), both of which indicate a decrease in muscle oxidative capacity.

### Short-term high-dose statin therapy reduces skeletal muscle mitochondrial respiratory function.

**Figure 2:**
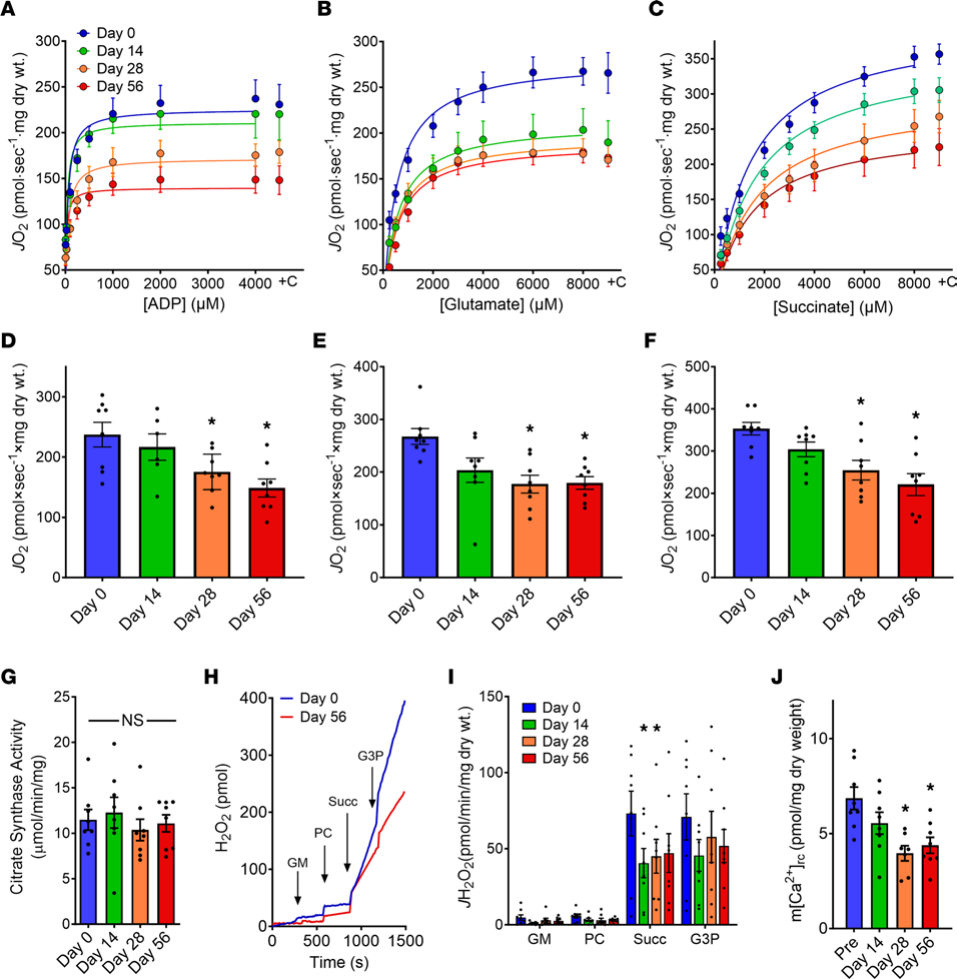
Short-term statin therapy progressively decreases skeletal muscle mitochondrial respiratory capacity. Permeabilized myofibers were prepared from skeletal muscle biopsies of the vastus lateralis,and mitochondrial function was measured by high-resolution respirometry prior to and after days 14, 28, and 56 of high-dose (80 mg/d) atorvastatin therapy. (**A**) ADP titration in the presence of 5 mM glutamate and 2 mM malate. (**B**) Glutamate titration in the presence of 2 mM malate and 4 mM ADP. (**C**) Succinate titration in the presence of 4 mM ADP and 10 μM rotenone. All protocols ended with a final addition of cytochrome c (+C) to check for mitochondrial integrity. (**D**–**F**) Maximal ADP-stimulated respiration from corresponding graphs in **A**–**C**. (**G**) Citrate synthase activity measured in whole muscle homogenates as an index of mitochondrial content. (**H**) Representative trace of mitochondrial H_2_O_2_ emission from permeabilized fiber bundles in response to sequential additions of 2 mM glutamate + 1 mM malate, 25 μM palmitoyl–L-carnitine, 10 mM succinate, and 10 mM glycerol-3-phosphate. (**I**) Mitochondrial H_2_O_2_ emitting rates (*J*H_2_O_2_) calculated from **H**. (**J**) Mitochondrial calcium retention capacity measured in permeabilized myofibers using fluorophore Calcium Green in the presence of 10 mM glutamate, 2 mM malate, and 0.2 mM ADP. Respiration was clamped with 5 mM 2-deoxyglucose and 2 U/mL hexokinase. Data are presented as mean ± SEM (*n* = 6–8 in **A** and **B**; *n* = 8 in **C**–**F**, **G**, and **I**; *n* = 7–8 in **J**). Data analyzed by repeated-measures 1-way ANOVA or mixed-effects analysis with Dunnett’s multiple-comparison test. *P < 0.05 was considered significant.

To directly assess the potential effect of statin therapy on skeletal muscle mitochondrial function, biopsies of the vastus lateralis muscle were taken prior to and 14, 28, and 56 days after initiating atorvastatin therapy. Permeabilized fiber bundles were prepared to measure mitochondrial respiratory kinetics and capacity using 3 different respiration protocols; in response to titration of ADP in the presence of saturating concentrations of complex I substrates (glutamate + malate; Figure 2A), in response to titration of glutamate in the presence of 2 mM malate and 4 mM ADP (Figure 2B), or in response to titration of the complex II substrate succinate in the presence of rotenone (complex I inhibitor to block reverse electron flow) and 4 mM ADP (Figure 2C). The first protocol tests the capacity/limitations of the entire oxidative phosphorylation (OxPhos) system, while the next 2 protocols provide insight regarding potential changes in the capacity/limitations at complex I or complex II of the electron transport system (ETS), respectively. All 3 protocols revealed a similar outcome — a progressive decline in the maximal ADP-stimulated rate of oxygen consumption (*J*O_2_) over the 56 days of atorvastatin treatment, resulting in a 30%–38% decrease in skeletal muscle mitochondrial OxPhos capacity (Figure 2, D–F). The apparent Km (i.e., the concentration of ADP or substrate eliciting 50% of maximal oxygen consumption; Figure 2, A–C) was unaffected in all protocols, indicating similar sensitivities of the OxPhos system to ADP and substrates. Addition of the mitochondrial uncoupler FCCP at the end of each titration protocol did not restore *J*O_2_ to prestatin treatment levels (i.e., day 0, not shown), indicating the statin-induced decrease in OxPhos capacity was not due to inhibition of ATP synthase. *J*O_2_ was also not restored by addition of cytochrome c (Figure 2, A–C), confirming that the integrity of the outer mitochondrial membrane remained intact with statin treatment. Moreover, neither citrate synthase activity (Figure 2G) nor expression of the ETS protein complexes (Supplemental Figure 2) were affected by statin therapy, indicating a decrease in mitochondrial content did not account for the decrease in OxPhos capacity. Atorvastatin therapy did not affect the mitochondrial H_2_O_2_ emitting potential except during respiration supported by succinate where it was decreased (Figure 2, H and I). Finally, statin treatment induced a decrease in mitochondrial calcium retention capacity (Figure 2J), indicative of an increase in sensitivity of the mitochondrial permeability transition pore to calcium overload.

### Acute in vitro exposure to statins decreases mitochondrial respiratory function.

To determine if atorvastatin directly affects skeletal muscle mitochondrial function, muscle biopsies were obtained after an overnight fast from a separate group of overweight but healthy female patients (ages 30–39, mean BMI = 33.8 kg/m^2^, good liver function, no statin history; Supplemental Figure 3A). Permeabilized fiber bundles were exposed to either vehicle or atorvastatin (10 μM) for 10 minutes prior to and throughout the respirometry protocol. Similar to atorvastatin treatment in vivo, exposure to atorvastatin in vitro reduced ADP-stimulated *J*O_2_ during titration of either complex I (Supplemental Figure 3B) or complex II (Supplemental Figure 3C) substrates.

### Mitochondrial complex IV is extremely sensitive to atorvastatin-induced inhibition.

**Figure 3:**
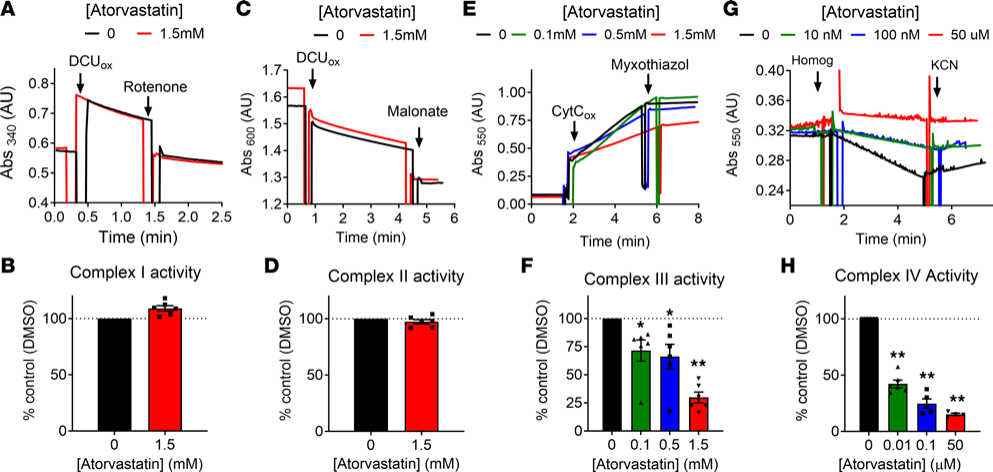
Acute statin exposure inhibits the activity of mitochondrial complex IV at low nanomolar concentrations in human skeletal muscle. Specific activities of mitochondrial ETS complexes were assessed spectrophotometrically from skeletal muscle lysates obtained from human participants in the presence of varying concentrations of atorvastatin. (**A**, **C**, **E**, and **G**) Representative traces. (**B**, **D**, **F**, and **H**) Quantification of complex I–IV specific activities, expressed as percent relative to DMSO treatment. Data are presented as mean ± SEM (*n* = 6 in **A**–**F**; *n* = 4–6 in **G** and **H**). Data analyzed by 1-way ANOVA with Sidak’s multiple-comparison test. *P < 0.05, **P < 0.01.

To explore the potential mechanisms by which atorvastatin inhibits mitochondrial function, steady-state maximal activity assays were performed for each of the 4 enzyme complexes in the ETS in the presence of increasing atorvastatin concentration using whole-muscle homogenates prepared from frozen portions of each patient’s baseline (i.e., day 0) muscle biopsy. Relative to vehicle, atorvastatin up to millimolar concentrations did not alter the specific activities of complex I (Figure 3, A and B) or complex II (Figure 3, C and D) of the ETS. Complex III activity was dose-dependently inhibited by atorvastatin, reaching approximately 70% inhibition relative to vehicle in the presence of 1.5 mM atorvastatin (Figure 3, E and F). Strikingly, atorvastatin inhibited complex IV activity at low nanomolar concentrations (~50% inhibition at 10 nM; Figure 3, G and H), reflecting a high sensitivity of complex IV to atorvastatin-induced inhibition.

To determine the extent to which atorvastatin accumulates in muscle in vivo, liquid chromatography with tandem mass spectrometry (LC-MS/MS) analysis was performed on muscle samples across all time points. Unfortunately, the tissue remaining for most of the samples was insufficient for analysis. Of the small subset of samples with sufficient tissue remaining (2 × 14 d, 1 × 28 d, 2 × 56 d), quantification of atorvastatin ranged from 2 to 23 ng per gram of muscle. Assuming a water content in muscle of approximately 0.75 mL/g and an even distribution within that volume, muscle atorvastatin concentration ranged from approximately 5 to 39 nM, well within the concentration required to inhibit complex IV activity. Surprisingly, even in this small number of samples, the statin-induced decrease in maximal mitochondrial ADP–stimulated *J*O_2_ correlated strongly with atorvastatin concentration (*R*^2^ = 0.9034) (Supplemental Figure 4).

## Discussion

The prevention of ASCVD remains a critical pillar of global medicine. Statins have been widely advocated by clinicians since early reports of their efficacy for the prevention of ASCVD (45–48). However, statins are not without risk; the most common side effect is some level of statin-induced myopathy (22, 31, 49–55). While the reported incidence of severe myopathy is relatively low, mild symptoms such as fatigue, muscle weakness, and tiredness are common. The goal of this study was to elucidate the potential mechanisms by which statins may induce muscle weakness/fatigue by using a combination of in vivo, in situ, and in vitro experimental approaches to assess whole-body aerobic capacity, skeletal muscle oxidative capacity, and mitochondrial respiratory capacity in skeletal muscle. Only high-intensity statin therapy was studied because it is most effective at lowering disease risk (56–58) and, in North America and other regions, is recommended for patients with a history of, or a high risk for, ASCVD (1, 59, 60). Within only 8 weeks of initiating 80 mg/d of atorvastatin, we report 2 main findings: (a) in skeletal muscle, postexercise recovery kinetics of mVO_2_ in vivo, as well as mitochondrial respiratory capacity in situ, were reduced by approximately 25% and approximately 30%–38%, respectively, and (b) at the level of individual respiratory chain complexes, direct in vitro exposure to micromolar concentrations of atorvastatin (i.e., 100 μM) inhibited complex III (<25%), whereas low nanomolar concentrations of atorvastatin (i.e., 10 nM) were sufficient to inhibit complex IV by > 50%.

The magnitude at which mitochondrial respiratory capacity in skeletal muscle decreased in response to 8 weeks of high-intensity atorvastatin treatment was striking. In cross-sectional studies, lower muscle mitochondrial respiratory capacity has been reported in statin-treated patients relative to matched controls (25, 35, 61). The reported effect was less than in the present study, but cross-sectional studies typically include many patients taking different types of statins over a wide range of doses and durations. In the only other study to directly examine the potential effect of statin treatment on mitochondrial respiratory capacity in muscle biopsy tissue, Asping and coworkers (36) found no difference in mitochondrial function in statin-naive patients after 2 weeks of high-dose statin treatment (simvastatin 80 mg/d or atorvastatin 40 mg/d). In the present study, muscle mitochondrial respiratory capacity was not reduced 2 weeks after initiating atorvastatin treatment but then did reach statistical significance at the 4- and 8-week marks. Decreased muscle oxidative capacity (12%), as assessed by ^31^P magnetic resonance imaging, has also been reported in patients after 4 weeks of atorvastatin therapy (80 mg/d) (62), in line with the approximately 25% reduction in in vivo muscle oxidative capacity measured by NIRS in the present study after 8 weeks of atorvastatin therapy. Collectively, these studies provide evidence that high-intensity statin therapy progressively decreases muscle respiratory capacity.

The potential effect of statins on aerobic capacity at the whole-body level is less clear. In the present study, average VO_2_ max was not significantly different after 8 weeks of statin therapy. However, 5 of 8 patients showed an appreciable decline (–4.3% to –16.5 %), whereas 3 showed virtually no change (Figure 1B). A prior open-label trial also found no change in VO_2_ max in 10 hyperlipidemic patients after 12 weeks of simvastatin (80 mg/d) treatment (63). Aerobic capacity in relation to statin treatment has also been assessed in 2 cross-sectional studies. In the LIFESTAT study, no difference in aerobic capacity was found between age (40–70 years) and BMI–matched controls (*n* = 20) and patients (*n* = 64) on statin therapy (minimum 40 mg/d simvastatin) for at least 3 months. However, in a much larger cohort study of 3,500 patients, statin usage was associated with significantly lower VO_2_ peak in males but not females (64). The gradual loss of aerobic capacity with aging is widely regarded as the single strongest predictor of all-cause mortality (65–68). Collectively, these findings suggest that additional larger-scale trials are needed to determine if statins adversely affect whole-body aerobic capacity.

Regular physical activity helps to preserve mitochondrial function and aerobic capacity, decreasing mortality risk and improving quality of life (69–74). For patients with ASCVD, mortality risk reduction is best achieved when statin therapy is combined with regular physical activity (75). However, physical activity has been reported to exacerbate statin-induced myalgia (20), particularly with higher-intensity exercise (23, 24) and/or in patients who are already symptomatic (25), although it is important to note that other studies have not found a relationship between exercise and statin-induced myalgia (76, 77). Previous studies have also found that statins interfere with the normal adaptive responses to aerobic exercise training by attenuating exercise training-induced increases in skeletal muscle mitochondrial content and overall cardiorespiratory fitness (26–28). However, 2 other studies have failed to confirm these findings, instead providing evidence that statins do not inhibit the adaptive responses to exercise training (62, 78). These discrepant outcomes have been discussed in recent reviews and are likely due to differences in dose and type of statin, the duration of therapy, and the order/duration in which statin therapy and/or exercise were initiated (i.e., concurrently versus one preceding the other) (79, 80). In addition, genetic polymorphisms influence statin catabolism and, thus, circulating statin levels and skeletal muscle exposure (81). The potential influence of genetic polymorphisms has not been examined in relation to skeletal muscle mitochondrial content/function or aerobic capacity during statin therapy, nor the interplay between statins and exercise. Thus, how statin dose, type, duration, exercise training, age, polymorphisms, and fitness level interact to affect ASCVD risk reduction, mitochondrial function, aerobic capacity, myalgia, and overall mortality remains to be thoroughly defined. The present findings do, however, point to the potential for progressive deterioration of skeletal muscle mitochondrial function with high-intensity statin therapy, even in relatively young healthy but overweight individuals, thus emphasizing the importance of carefully assessing the risk-to-benefit ratio over time for patients initiating statin therapy. Provided myalgia is not exacerbated, it is also possible that implementing a regular exercise program with high-dose statin therapy may benefit patients by at least partially counteracting statin-induced decreases in mitochondrial function.

Several mechanisms associated with mitochondrial function are thought to contribute to the development of statin-induced myopathy, including altered calcium homeostasis (32, 82), decreased ubiquinone content (Coenzyme Q10) (83) and respiratory function (29–31, 34, 82), reduced mitochondrial DNA content (84), and induced muscle atrophy (85). A recent report has also provided evidence that the lactone form of several different statins (100 μM) directly inhibits complex III, with no effect on complex IV, in mitochondria from C2C12 myoblasts (31). This same study also reported decreased complex III (~15%), but not complex IV, activity in a cross-sectional comparison of muscle biopsies from control patients and patients with statin-induced myopathies (31). In the present study, direct exposure of mitochondrial extracts from human skeletal muscle to atorvastatin, beginning at 100 μM, also inhibited complex III activity. However, complex IV activity was inhibited by > 50% at low nM concentrations of atorvastatin (i.e., 1 × 10^4^ lower than that required to inhibit complex III). What accounts for the discrepancy between the 2 studies is unclear but may reflect differences in model systems (i.e., C2C12 myoblasts versus human mitochondrial extracts; ref. 86) and/or human study design (i.e., cross-sectional versus repeated measures). Complex IV activity has been reported to be lower in skeletal muscle mitochondria of long-term statin users that are symptomatic of statin-induced myopathy relative to nonstatin users (25). Although more research is needed, the finding that complex IV was inhibited in vitro at atorvastatin concentrations present in skeletal muscle of patients in vivo (i.e., low nM) (Figure 3H and Supplemental Figure 4) provides another plausible mechanism by which atorvastatin may decrease skeletal muscle mitochondrial respiratory capacity.

There are several limitations to the study. This was not a randomized clinical trial with a control group but rather a small study using a repeated measures design. To minimize potential confounding factors, patient recruitment was deliberately restricted to sedentary and modestly overweight but otherwise relatively young and healthy individuals. The study did not include patients with health indications that typically prompt statin prescription, including hyperlipidemia and ASCVD; therefore, it is not known whether such patients will experience similar loss of skeletal muscle mitochondrial function. Only 1 type of statin was tested, and it is not known whether other types, particularly lipid soluble versus nonlipid soluble, will elicit similar outcomes. Muscle strength was not formally assessed; thus, its potential relationship to the reduction in mitochondrial respiration could not be determined. The correlation between muscle atorvastatin concentration and change in maximal mitochondrial ADP–stimulated oxygen consumption (Supplemental Figure 4) should be interpreted with caution, given the limited number of samples available. Finally, only 1 dose of atorvastatin was tested, and it remains to be determined how the interplay between statin dose, therapy duration, and patient age/health status/activity level may affect the susceptibility of skeletal muscle to loss of mitochondrial function.

In summary, the present findings provide evidence that high-dose atorvastatin therapy elicits a progressive and marked decline in skeletal muscle mitochondrial functional capacity in humans, and that direct inhibition of mitochondrial complex IV may be a primary mechanism of action. Reduced skeletal muscle mitochondrial function may contribute to a decrease in whole-body aerobic capacity, although more data are needed to test this hypothesis in the context of statin therapy. The findings highlight the need for longer-term, large cohort studies to further define the potential interactions between statin type/dose/duration and patient health status on skeletal muscle mitochondrial function and overall aerobic capacity.

## Methods

### Sex as a biological variable.

Both male and female patients participated in this study, and similar findings are reported for both sexes. Other than where noted, no sex differences were detected and, as such, all data were combined.

### Reagents.

Unless otherwise noted, all chemicals were purchased from Sigma-Aldrich and of the highest purity available.

### Participants.

Eight overweight sedentary adults (4 male, 4 female) were recruited to participate in the study. Aside from elevated BMI, participants were otherwise healthy, nonsmokers, and not taking any medications known to alter metabolism. Physical activity was quantified via the International Physical Activity Questionnaire (IPAQ) at baseline (87). All patients were classified as inactive or minimally active and instructed to maintain their normal activity levels during the 8-week study. All female participants were enrolled for baseline testing on the first day of their menstrual cycle. Clinical and physical characteristics are provided in Table 1.

### Study design.

Participants completed baseline testing prior to initiating the study; this included determination of whole-body VO_2_ max, in vivo muscle oxidative capacity via NIRS, and IVGTT following an overnight fast. Following at least 2 days of rest, participants again reported after an overnight fast and a percutaneous biopsy was obtained from the vastus lateralis muscle. Participants then began the atorvastatin (80 mg/day) treatment for 56 consecutive days with instructions to take the pills in the morning upon waking and to maintain their normal diet and activity levels for the duration of the study. Additional muscle biopsies were obtained on the mornings of days 14, 28, and 56. Participants were asked at each visit, and instructed to report at any time, whether they experienced any muscle-related symptoms (e.g., pain, weakness, discomfort) apart from the biopsies. An IVGTT was repeated on day 56 after the muscle biopsy. NIRS and VO_2_ max tests were repeated within 5–7 days after the final biopsy, with patients remaining on the statin regimen.

### VO_2_ max.

Peak oxygen consumption rate was determined by indirect calorimetry (Parvo Medics’ TrueOne 2400 metabolic cart) on a motor driven treadmill (Cardiac Science) using a modified Bruce treadmill protocol. After an initial 3-minute warm-up at 2.0 mph and 0% grade, treadmill velocity was increased to 3.0 mph. Thereafter, the grade was increased by 2% every 2 minutes until volitional fatigue. At least 2 of the following criteria were used to validate achieving VO_2_ max; evidence of plateau in VO_2_ (<50% of slope of submaximal work rate × VO_2_), respiratory exchange ratio ≥ 1.05, heart rate ≥ 95% of the age-predicted maximum (220 – age), and/or 2 minutes posttest blood lactate > 6.0 nmol/L. The breakdown of criteria outcomes for each patient’s pre- and posttraining VO_2_ test is provided in the Supplemental Data Values file.

### In vivo assessment of mitochondrial function via NIRS.

Skeletal muscle mitochondrial respiratory capacity was assessed in vivo using NIRS as previously described (41). NIRS data were acquired using a frequency-domain device (OxiplexTS, ISS) equipped with 8 infrared diode lasers (4 emitting at 691 nm and 4 at 830 nm) and a detector. In the current study, we employed a single-channel (i.e., probe) setup with emitter-detector distances of 2.5, 3.0, 3.5, and 4.0 cm. Data were collected at 4 Hz. The NIRS probe was positioned longitudinally on the belly of the vastus lateralis muscle of the right leg approximately 10 cm above the patella and secured with double-sided adhesive tape and Velcro straps around the thigh. The NIRS device was calibrated prior to each test using a phantom with known optical properties after a warm-up period of at least 30 minutes. A blood pressure cuff (Hokanson SC-10D or SC-10L) was placed proximal to the NIRS probe as high as anatomically possible on the thigh. The blood pressure cuff was controlled with a rapid-inflation system (Hokanson E20, D.E. Hokanson Inc.) set to a pressure of > 250 mmHg and powered with a 15-gallon air compressor (Model D55168, Dewalt). Skin and adipose tissue thickness was measured at the site of the NIRS probe using skinfold calipers (Lange). Participants performed a short-duration (~10–20 seconds) isometric contraction of the quadriceps muscle group to increase mVO_2_. Upon relaxation, the recovery kinetics of mVO_2_ were measured using a series of transient arterial occlusions, and data were fitted to a single exponential function, where *end* is the mVO_2_ immediately following exercise, *delta* is the change in mVO_2_ from rest to end-exercise, *k* is the rate constant, and τ is the time constant (1/*k*). The rate constant was used as an index of mitochondrial respiratory capacity as previously described (44). The exercise and occlusion procedure was performed twice, and the results were averaged. NIRS data were analyzed using custom-written routines in Matlab (Mathworks).

### Fasting blood draw and IVGTT.

Upon arrival after an overnight fast, participants rested in bed for 20 minutes. A catheter was placed in the antecubital vein, and a baseline blood draw was obtained for routine laboratory testing of metabolic and liver function panels. Immediately following baseline blood sampling, an IVGTT was performed as previously described (88).

### Skeletal muscle biopsies.

Percutaneous muscle biopsy samples were collected from the vastus lateralis muscle. After cleansing the skin with a povidone-iodine swab (or chlorhexidine gluconate for patients allergic to iodine/shellfish), the biopsy sight was then anesthetized with 5 cc of lidocaine. A small incision was made with a scalpel, and the muscle biopsy sample was aspirated through a 5 mm Bergström needle. Part of the muscle biopsy sample was immediately flash frozen in liquid nitrogen and stored at –80°C until subsequent analysis. Remaining portions of the muscle biopsy were used for mitochondrial function assessment (~10–20 mg).

### Preparation of permeabilized fiber bundles.

Approximately 100–150 mg of skeletal muscle was obtained from the vastus lateralis muscle by percutaneous muscle biopsy under local anesthetic (1% lidocane). A portion of each muscle sample was immediately placed in ice-cold buffer X (50 mM K-MES, 7.23 mM K_2_EGTA, 2.77 mM CaK_2_EGTA, 20 mM imidazole, 20 mM taurine, 5.7 mM ATP, 14.3 mM phosphocreatine, and 6.56 mM MgCl_2_-6H_2_O [pH 7.1]) for preparation of permeabilized fiber bundles as previously described (89). Fiber bundles were separated along their longitudinal axis using needle-tipped forceps under magnification (MX6 Stereoscope, Leica Microsystems), permeabilized with saponin (30 μg/mL) for 30 minutes at 4°C, and subsequently washed in cold buffer Z (105 mM K-MES, 30 mM KCl, 1 mM EGTA, 10 mM K_2_HPO_4_, 5 mM MgCl_2_-6H_2_O, 0.5 mg/mL BSA [pH 7.1]) for approximately 20 minutes until analysis. At the conclusion of each experiment, permeabilized fiber bundles (PmFBs) were washed in double-distilled H_2_O to remove salts, freeze-dried (Labconco), and weighed for data normalization. Typical fiber bundle sizes were 0.2–0.6 mg dry weight.

### Mitochondrial respiration.

High-resolution O_2_ consumption was measured in permeabilized fiber bundles in 2 mL of buffer Z containing 20 mM creatine and 25 μM blebbistatin to inhibit contraction (89) using the OROBOROS Oxygraph-2k (Oroboros Instruments). Polarographic O_2_ measurements were acquired at 2-second intervals with the steady state rate of respiration calculated from a minimum of 40 data points and expressed as pmol/sec/mg dry weight. All respiration measurements were conducted at 37°C and a working range [O_2_] of approximately 350–200 μM.

### Mitochondrial H_2_O_2_ emission.

Mitochondrial H_2_O_2_ emission rate was measured fluorometrically at 37°C via the Amplex Ultra Red (10 μM)/horseradish peroxidase (HRP: 3 U/mL) detection system (excitation/emission [Ex:Em], 565:600) in response to sequential additions of 2 mM glutamate + 1 mM malate, 25 μM palmitoyl–L-carnitine, 10 mM succinate, and 10 mM glycerol-3-phosphate (90).

### Mitochondrial calcium retention capacity.

To determine susceptibility to opening of the mitochondrial permeability transition pore, permeabilized fiber bundles were exposed to progressively increasing calcium load in the presence of (in mM): 5 malate, 10 glutamate, and 0.02 ADP. Changes in extramitochondrial calcium concentration were monitored fluorometrically using Calcium Green (1 μM; Ex:EM, 506:532 nm; Invitrogen) per the manufacturer’s instructions. All experiments were run at 37°C in Buffer Z containing 2 U/mL hexokinase and 5 mM 2-deoxyglucose to clamp respiration.

### Specific activity of mitochondrial OxPhos complexes.

Frozen portions from muscle biopsies were homogenized in 0.3M sucrose, 10 mM HEPES, and 1 mM EGTA on ice (adapted from ref. 91), and total protein concentration was determined using the BCA protein assay kit (Invitrogen). Citrate synthase activity was measured using a standard assay kit (MilliporeSigma, CS0720). Specific activities of each individual ETS complex were determined spectrophotometrically as previously described (92). Briefly, aliquots of skeletal muscle lysates were diluted in hypotonic medium (25 mM K_2_HPO_4_, 5.3 mM MgCl_2_ [pH 7.2]) and further subjected to 3–4 freeze-thaw cycles. Complex I activity was determined in 5 mM Tris, 0.5 mg/mL BSA, 24 μM KCN, 0.4 μM antimycin A (pH 8), following the oxidation of NADH (0.8 mM) at 340 nm (ε_340_ = 6,220/M/cm) for 3 minutes using oxidized decyl-ubiquinone (DCU_ox_; 50 μM) as the electron acceptor. Rotenone (4 μM) was added to measure rotenone-sensitive NADH-DCU oxidoreductase activity. Complex II activity was measured in 10 mM KH_2_PO_4_, 2 mM EDTA, and 1 mg/mL BSA in the presence of 10 mM succinate (SQR medium), following the reduction of dichlorophenolindophenol (80 μM) at 600 nm (ε_340_ = 19,100/M/cm) for 3 minutes, using DCU_ox_ as the electron acceptor. The reaction was inhibited by the addition of the competitive substrate malonate (10 mM). Complex III activity was measured in SQR medium supplemented with 200 μM ATP and 240 μM KCN, using DCU_red_ (80 mM) as an electron donor and oxidized cytochrome c as acceptor (40 μM). The reaction was followed by measuring the reduction of cytochrome c at 550 nm (ε_340_ = 18500/M/cm) for 3 minutes and was then inhibited by the addition of 0.5 μM myxothiazol. Complex IV activity was measured in 10 mM KH_2_PO_4_ (pH 6.5), 0.25 M sucrose, 1 mg/mL BSA, and 10 μM of reduced cytochrome c. Lauryl maltoside (2.5 mM) was added to permeabilize the external mitochondrial membrane, and the rate of cytochrome c oxidation was followed measuring the decrease in absorbance at 550 nm.

### Immunoblotting.

Frozen skeletal muscle samples from the biopsies were homogenized in radioimmunoprecipitation buffer supplemented with protease inhibitors (Roche). Immunoblotting for the mitochondrial OxPhos complexes was performed using the total OxPhos antibody cocktail (Abcam, ab110411) at 1:1,000. Bands were quantified by densitometry using ImageJ (NIH), and relative intensities were normalized to GAPDH (Abcam, ab110413) at 1:1,000.

### Measurement of atorvastatin by LC-MS/MS.

Frozen skeletal muscle samples obtained from the biopsies were lysed using a Tissuelyser (3 × 20 cycles/sec for 2 minutes) in 1 mL of 90:10 acetonitrile/water with 0.1% formic acid. Samples were then centrifuged for 10 minutes at 15,000*g* at 4°C. The pellet was resuspended in homogenization buffer, spiked with an internal standard mix of atorvastatin, and evaporated to dryness under nitrogen gas. Samples were resuspended in 65:35 acetonitrile/water for injection in LC-MS/MS.

### Statistics.

Data are presented as mean ± SEM unless otherwise specified. Analyses performed included repeated-measures 1-way ANOVA or mixed-effects analysis (in the case of 1 or more missing values) with Dunnett’s multiple-comparison test, ordinary 1-way ANOVA with Sidak’s multiple-comparison test, and 2-tailed paired *t* tests. Statistical analyses were performed with GraphPad Prism (GraphPad Software). Statistical significance was accepted when *P* < 0.05.

### Study approval.

All procedures were approved by the IRB for patients at East Carolina University and carried out in accordance with the Declaration of Helsinki. All participants gave written informed consent prior to enrollment. Patients participating in the study were recruited prior to release of the NIH Policy on the Dissemination of NIH-Funded Clinical Trial Information. Therefore, the study is not registered through ClinicalTrials.gov.

### Data availability.

All supporting raw data and statistical analyses are provided in the Supporting Data Values file.

## Author contributions

TER, CTL, and PDN designed the study. TER, PMB, and AHC recruited the patients and collected all patient samples and clinical data. TER, MJT, CAS, and CDS performed sample analyses. EMM and JPT performed LC-MS/MS analysis and data interpretation. TER, MJT, CTL, and PDN conducted statistical analysis, data interpretation, and figure generation. TER, CTL, and PDN supervised the study. TER and PDN wrote the manuscript. All authors contributed to the draft edits and have approved the final version of the manuscript.

## Supplementary Material

Supplemental data

ICMJE disclosure forms

Unedited blot and gel images

Supporting data values

## Figures and Tables

**Table 1:**
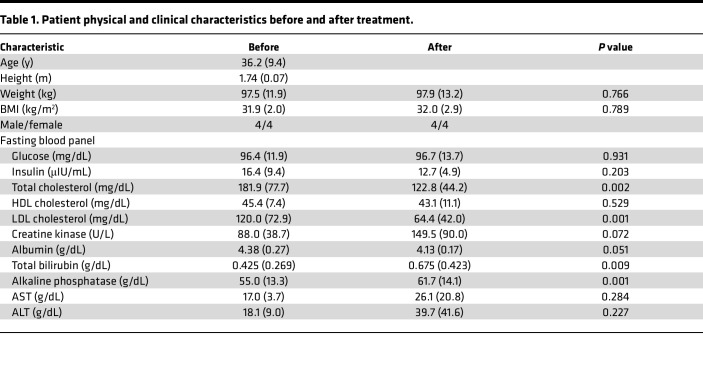
Patient physical and clinical characteristics before and after treatment.

## Version 1 02/22/2024

Electronic publication
