# Supplementary material for: High-dose atorvastatin therapy progressively decreases skeletal muscle mitochondrial respiratory capacity in humans
Source: JCI Insight. 2024 Feb 22;9(4):e174125. doi: 10.1172/jci.insight.174125 (PMC10967389; doi:10.1172/jci.insight.174125)
Supplement: Supplemental data [file jciinsight-9-174125-s200.pdf]

High Dose Atorvastatin Therapy Progressively Decreases Skeletal Muscle Mitochondrial Respiratory Capacity in Humans

Terence E. Ryan[1,2], Maria J. Torres[1,4], Chien-Te Lin[1,2], Angela H. Clark[1], Patricia M. Brophy[1], Cheryl A. Smith[1,2], Cody D. Smith[1,2], E. Matthew Morris[5], John P. Thyfault[5], P. Darrell Neufer[1,2,3]

[1]East Carolina Diabetes and Obesity Institute, [2]Department of Physiology, [3]Department of Biochemistry and Molecular Biology, Brody School of Medicine, [4]Department of Kinesiology, East Carolina University, Greenville, NC, USA [5]Department of Molecular and Integrative Physiology, University of Kansas Medical Center, Kansas City, KS, USA

Supplemental Data

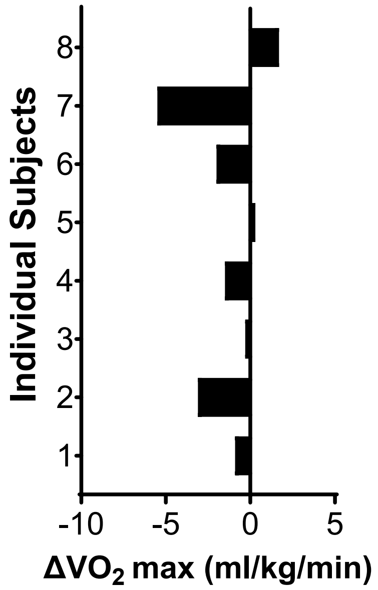

Figure S1. Individual absolute changes in VO_2 max after 56 days of statin therapy.

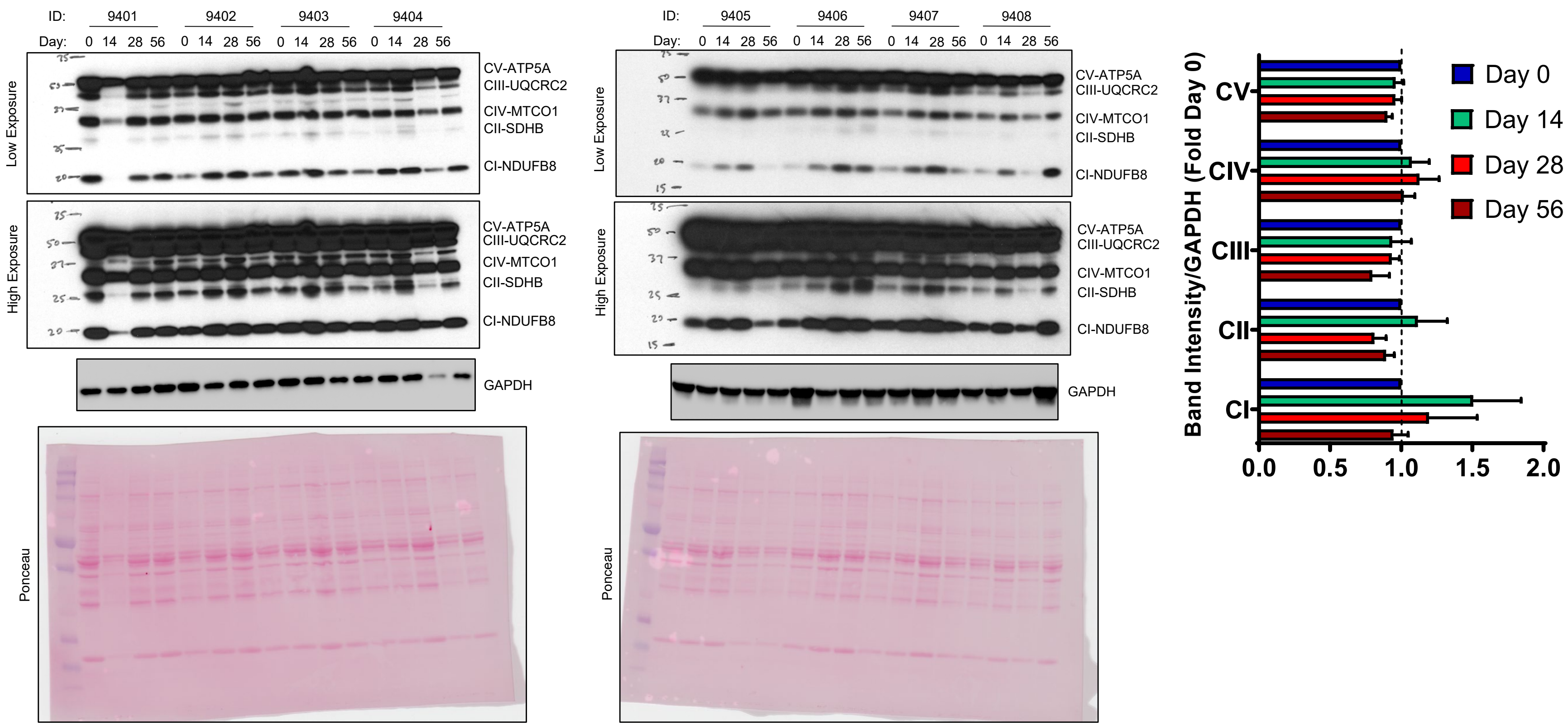

Figure S2. Electron transport chain complex expression in skeletal muscle. Western blot images of mitochondrial electron transport complexes I-V, as well as GAPDH, from skeletal muscle homogenates of all subjects (9401-9408) prior to (day 0) and 14d, 21d, and 56d after initiating atorvastatin (80 mg/ml) therapy. Right panel shows summary data. N=8.

A.

Characteristic	
Age (y)	35.9 (3.9)
Height (m)	1.65 (0.06)
Weight (kg)	93.8 (17.4)
BMI (kg/m^2)	33.8 (6.0)
Male/Female	0/6
Fasting Blood Panel	
Glucose (mg/dL)	94.0 (5.5)
Insulin (µIU/mL)	12.1 (5.0)
Total Cholesterol (mg/dL)	175.5 (16.5)
HDL Cholesterol (mg/dL)	52.7 (9.2)
LDL Cholesterol (mg/dL)	106.0 (16.4)
Creatine Kinase (U/L)	88.6 (34.5)
Albumin (g/dL)	4.07 (0.2)
Total Bilirubin (g/dL)	0.30 (0.09)
Alkaline Phosphatase (g/dL)	69.7 (25.2)
AST (g/dL)	13.5 (2.7)
ALT (g/dL)	10.5 (5.4)

B.

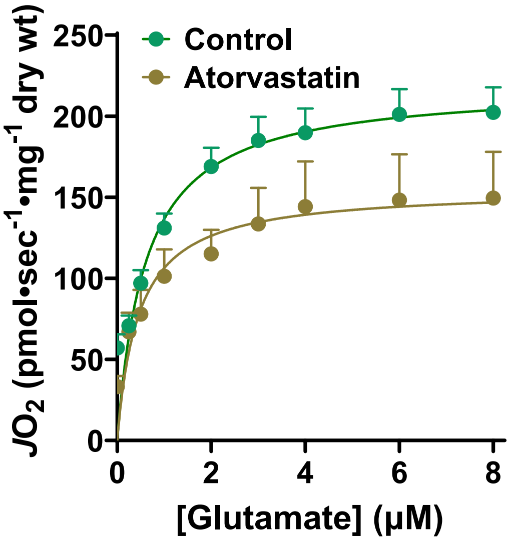

C.

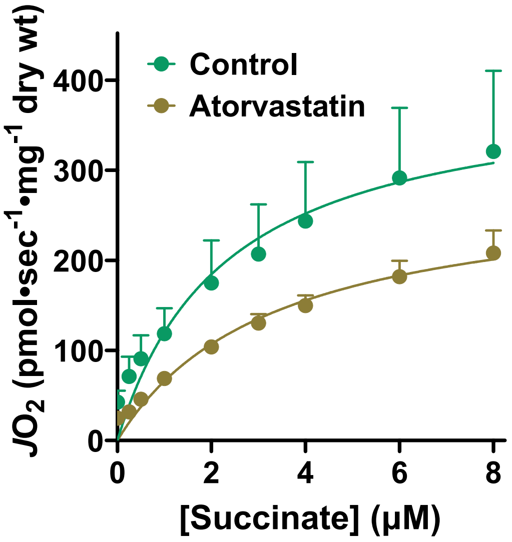

Figure S3. Acute in vitro exposure to atorvastatin decreases ADP-stimulated oxygen consumption in human permeabilized skeletal muscle fiber bundles. (A) Characteristics of a separate group of sedentary, overweight but otherwise healthy subjects not currently taking statins from whom fasting muscle biopsies were obtained for acute in vitro studies. (B-C)Permeabilized fiber bundles were exposed to atorvastatin (10 µM) for 10 min prior to and during titration with either glutamate plus malate (2 mM; B) or succinate plus rotenone (10 µM; C) in the presence of 4 mM ADP. Data are presented as means ± SEM, N=6.

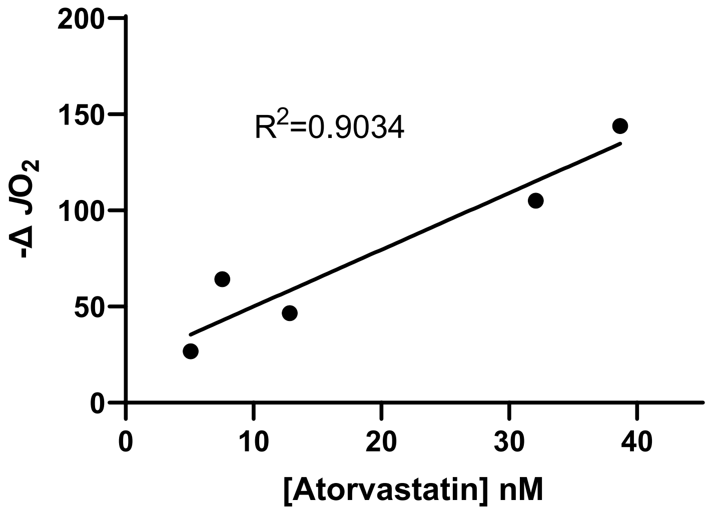

Figure S4. Correlation between statin-induced decrease in maximal mitochondrial ADP-stimulated JO_2 and atorvastatin concentration in skeletal muscle.
