# Supplementary material for: High-dose atorvastatin therapy progressively decreases skeletal muscle mitochondrial respiratory capacity in humans
Source: JCI Insight. 2024 Feb 22;9(4):e174125. doi: 10.1172/jci.insight.174125 (PMC10967389; doi:10.1172/jci.insight.174125)

Lane	Time Point	Sample ID
Gel #1		
1	Ladder-12uL	
2	day 0	9401
3	day 14	9401
4	day 28	9401
5	day 56	9401
6	day 0	9402
7	day 14	9402
8	day 28	9402
9	day 56	9402
10	day 0	9403
11	day 14	9403
12	day 28	9403
13	day 56	9403
14	day 0	9404
15	day 14	9404
16	day 28	9404
17	day 56	9404
18		
Gel # 2		
1	Ladder-12uL	
2	day 0	9405
3	day 14	9405
4	day 28	9405
5	day 56	9405
6	day 0	9406
7	day 14	9406
8	day 28	9406
9	day 56	9406
10	day 0	9407
11	day 14	9407
12	day 28	9407
13	day 56	9407
14	day 0	9408
15	day 14	9408
16	day 28	9408
17	day 56	9408
18		

Gel #1 Ponceau

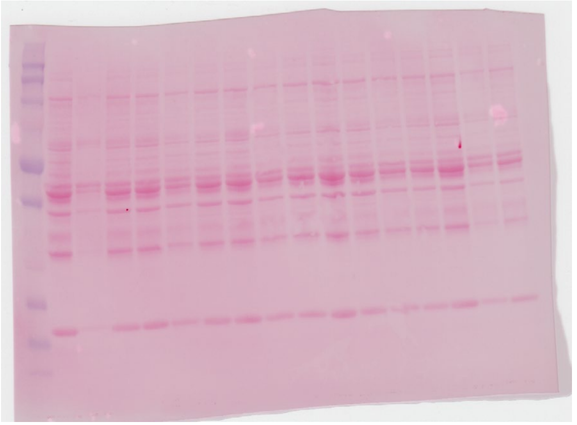

Gel #1 Total Oxphos low exposure

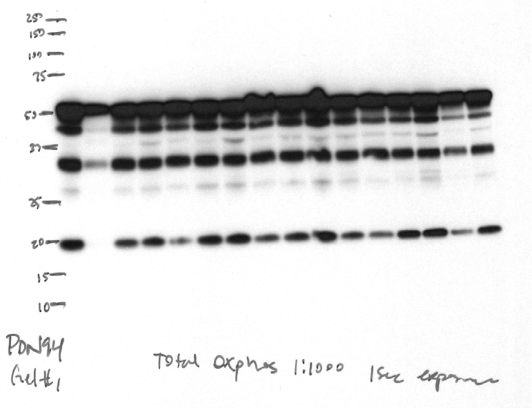

Gel #1 Total Oxphos high exposure

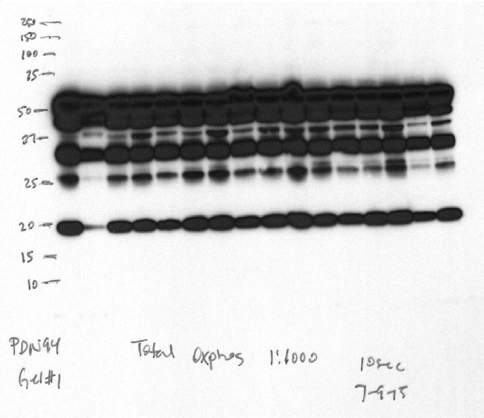

Gel #1 GAPDH

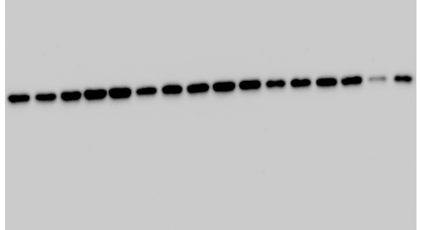

Gel #2 Ponceau

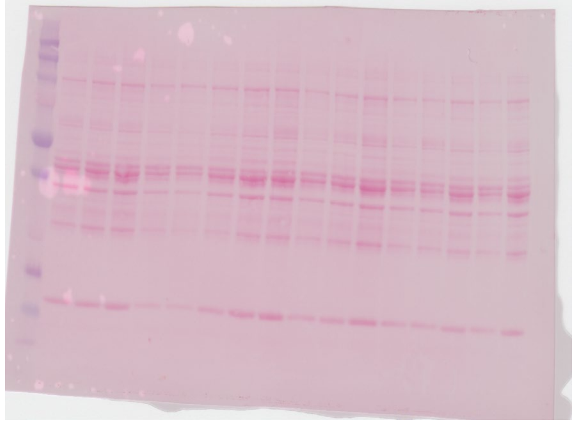

Gel #2 Total Oxphos low exposure

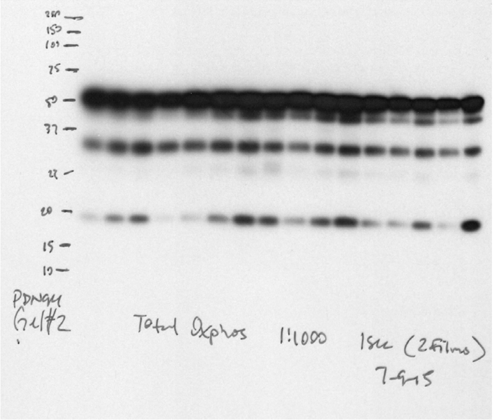

Gel #2 Total Oxphos high exposure

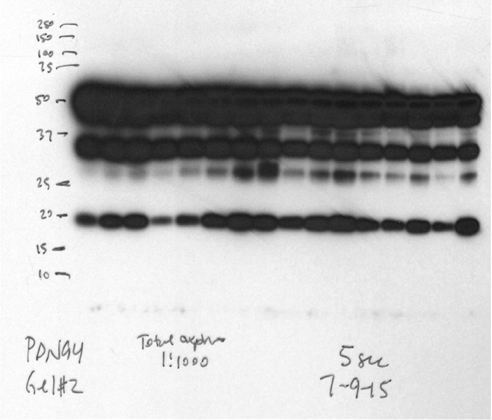

Gel #2 GAPDH

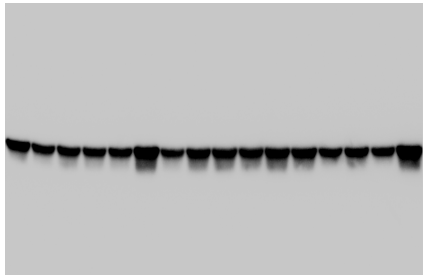

Supplement: Unedited blot and gel images [file jciinsight-9-174125-s202.pdf]
